# MIKC type MADS-box transcription factor LcSVP2 is involved in dormancy regulation of the terminal buds in evergreen perennial litchi (*Litchi chinensis* Sonn.)

**DOI:** 10.1093/hr/uhae150

**Published:** 2024-05-28

**Authors:** Meng-Meng Ma, Hui-Fen Zhang, Qi Tian, Hui-Cong Wang, Fang-Yi Zhang, Xue Tian, Ren-Fang Zeng, Xu-Ming Huang

## Abstract

SHORT VEGETATIVE PHASE (SVP), a member of the MADS-box transcription factor family, has been reported to regulate bud dormancy in deciduous perennial plants. Previously, three *LcSVPs* (*LcSVP1*, *LcSVP2* and *LcSVP3*) were identified from litchi genome, and *LcSVP2* was highly expressed in the terminal buds of litchi during growth cessation or dormancy stages and down-regulated during growth stages. In this study, the role of LcSVP2 in governing litchi bud dormancy was examined. *LcSVP2* was highly expressed in the shoots, especially in the terminal buds at growth cessation stage, whereas low expression was showed in roots, female flowers and seeds. LcSVP2 was found to be located in the nucleus and have transcription inhibitory activity. Overexpression of *LcSVP2* in *Arabidopsis thaliana* resulted in a later flowering phenotype compared to the wild-type control. Silencing *LcSVP2* in growing litchi terminal buds delayed re-entry of dormancy, resulting in significantly lower dormancy rate. The treatment also significantly up-regulated litchi *FLOWERING LOCUS T2* (*LcFT2*). Further study indicates that LcSVP2 interacts with an AP2-type transcription factor, SMALL ORGAN SIZE1 (LcSMOS1). Silencing *LcSMOS1* promoted budbreak and delayed bud dormancy. Abscisic acid (200 mg/L), which enforced bud dormancy, induced a short-term increase in the expression of *LcSVP2* and *LcSMOS1*. Our study reveals that LcSVP2 may play a crucial role, likely together with LcSMOS1, in dormancy onset of the terminal bud and may also serve as a flowering repressor in evergreen perennial litchi.

## Introduction

Dormancy is an important phase during plant growth and development. Three types of dormancy were reported previously. They are paradormancy, endodormancy, and ecodormancy [1]. Researchers believe that endodormancy is an approach for temperate deciduous trees to cope with harsh winter conditions [2]. Plants growing in a Mediterranean climate region always enter dormancy in summer to survive the extreme high temperature and dry environment [3]. However, for evergreen perennials such as litchi (*Litchi chinensis* Sonn.), dormancy of the terminal bud is not developed for survive harsh conditions, as it occurs even in favorable conditions throughout the intermittent growth of flushes [4]. As a part of internal flush growth rhythm, the dormancy of litchi terminal buds was defined as endodormancy by Zhang et al. [4].

The study on regulatory mechanisms of plant bud dormancy development is intriguing subject for researchers. In the recent decade, a lot of work has been done in studying of plant bud growth cessation and dormancy in various species. In woody species of poplar, related work has been meticulously studied [5]. In many deciduous fruit trees, shoot growth cessation and bud differentiation often occur in late spring and early summer, during which the day length becomes longer and the temperature becomes higher, while the bud dormancy usually happens in late autumn, when the temperature decreases and the day length becomes shorter [6]. However, the growth cessation and bud dormancy of some deciduous fruit trees is not strictly induced by short day. Previous studies showed that in some fruit trees, growth arrest was induced by low temperatures rather than short day conditions [7]. *Dormancy Associated MADS-box* (*DAM*) and *SHORT VEGETATIVE PHASE* (*SVP*)-like genes responsible for dormancy have been identified in temperate fruit trees of rosaceous species [8], and they play a crucial role in regulating growth cessation and bud set [9, 10]. In addition to rosaceous species, DAM or SVP-like genes have been reported to be involved in the dormancy cycle in other species such as leafy spurge and poplar [11–13].

The DAM proteins belong to MIKC^c^ type MADS-box family. These type family proteins contain four domains: MADS-box, K-box, I-box, and C-terminal domains [14]. *DAM* genes were also known as floral regulatory factor *SVP* or *AGAMOUS-LIKE 22*/*24* (*AGL22*/*24*) in *Arabidopsis thaliana*. SVP serves as a key factor in the development of *A. thaliana*, continually functioning as a suppressor flower development in the plant's vegetative stage [15, 16]. However, some studies suggested that the *SVP* or *DAM* genes have different functions in annual and perennial species, such as controlling the induction of floral and regulating the dormancy cycle. In a previous study, a total of 6 DAMs (*PpeDAM1* to *PpeDAM6*) were identified in peach [10]. Interestingly, it showed that *DAM* gene sequences are a highly homologous to *SVP* genes in *A. thaliana*, and sometimes in other plants, they were referred as *SVP-like* genes. Recent research suggested that *DAM* or *SVP* involved in the regulation of bud dormancy in temperate deciduous fruit trees [8]. A few downstream targets of DAM–SVP transcription factors have been identified, for instance *FLOWERING LOCUS T* (*FT*)*-like* genes, which are down-regulated by DAM–SVPs in the study of leafy spurge and pear [17, 18]. *NCED3*, a key gene involved in the biosynthesis of abscisic acid (ABA), is activated by PpDAM1 in pear [19]. Additionally, in hybrid aspen, a DAM/SVP-like gene (*SVL*) was revealed to suppress budbreak by promoting *NCED3* and ABA receptors genes’ expression [20]. These findings bring the understanding of dormancy or flowering regulation pathways involving DAM–SVPs.

Unlike temperate deciduous trees, most evergreen subtropical or tropical trees, such as citrus [21], mango [22], and litchi [23], usually undergo a number of flush growth cycles within a year. Litchi is an important evergreen fruit, which has complicated genetic background with 15 chromosomes and high heterozygosity, and has been cultivated in subtropical or tropical regions for thousands of years [24, 25]. However, shoot growth of litchi is intermittent, as the terminal bud alternates between growth and dormancy [23]. Dormancy of litchi is restricted to the terminal meristem in the shoot tip but not to the lateral meristem (cambium) as the stem thickening is continuous [23]. Although dormancy of litchi terminal bud can be enforced by adverse conditions such as drought and cold, the alternation between growth and dormancy in terminal buds occurs in growth-favorable conditions, suggesting the dormancy of the terminal litchi bud is induced by internal factors, among which phytohormones may play crucial role. In deciduous perennials, ABA is a major player in regulating the development and maintenance of bud dormancy [12, 26, 27]. However, there has been no report about roles of ABA in bud dormancy in evergreen litchi. Exogenous ethylene (ethephon) is effective to enforce and maintain terminal dormancy of litchi bud [28].

A lack of information can be obtained on the molecular mechanisms of bud dormancy development in evergreen perennials compared to deciduous perennials. A pioneer transcriptomic study revealed three *LcSVP* genes in litchi bud, and the expression pattern of *LcSVP2* indicated that it might be associated with the development and maintenance of bud dormancy [4]. Hu et al. [29] later provided indirect evidence showing that all the three *LcSVPs* identified by Zhang et al. [4] might serve as flowering repressors in litchi.

In this study, the functions of *LcSVP2* were further analyzed by ectopic overexpression in *A. thaliana* and transformation in litchi. To explore the pathway involving the regulation role of LcSVP2 in dormancy of litchi terminal bud, a yeast library was previously created from litchi terminal buds for screening potential proteins that interact with LcSVP2. An AP2-type transcription factor LcSMOS1 protein was screened to interact with LcSVP2. Additionally, the interaction between LcSMOS1 and LcSVP2 was further analyzed, and the role of LcSMOS1 in bud dormancy regulation was also explored.

## Results

### Identification and characterization analysis of litchi *LcSVP2* gene

**Figure 1:**
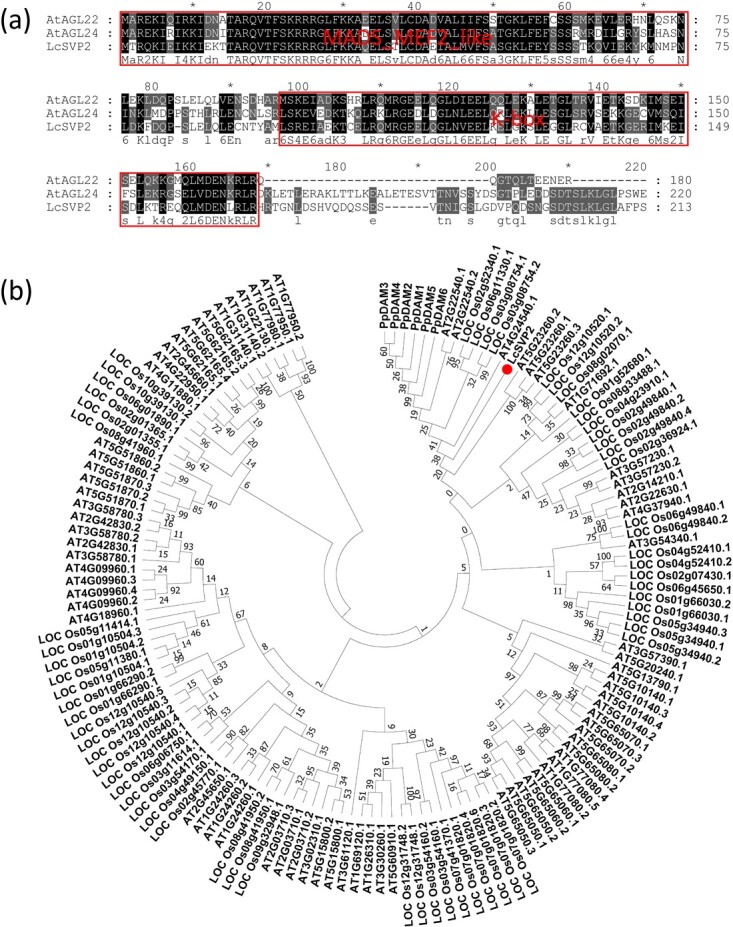
Homology analysis of LcSVP2. (a). Multiple sequence alignments. (b). Phylogenetic tree of SVP or DAM proteins from *Populus trichocarpa*, *Vitis vinifera*, *Pyrus bretschneideri*, *Malus domestica*, *Arabidopsis thaliana*, *Citrus sinensis*, *Prunus mume*, *Prunus persica*, *Dimocarpus longan*, *Nephelium lappaceum*, and *Litchi chinensis*. The alignment of amino acid sequences were created using ClustalX version 1.83 and the phylogenetic tree was created using MEGA 7.0 by the NJ method with 1000 bootstrap replicates

In our previous study, three *SVP-like* genes (*LcSVP1*, *LcSVP2*, and *LcSVP3*) were identified from RNA-sequencing of litchi shoot terminal buds at different stages, and based on the expression pattern, *LcSVP2* was suggested to be involved in dormancy onset as it was highly expressed in growth cessation stage (Stage 4) [4]. To explore the sequence characteristics of LcSVP2, a sequence alignment was performed with homologous proteins AGL22 and AGL24 of Arabidopsis (Fig. 1). The highly-conserved domains MADS-box and K-box were found among the LcSVP2 and AGL22/24, and the sizes of those proteins were distributed between 180 and 220 amino acids (Fig. 1a). Furthermore, the phylogenetic relationships of LcSVP2 and MIKC type MADS-box family members from Arabidopsis and *O. sativa*, along with six DAM proteins of *P. persica,* were analyzed using MEGA7.0 based on 1000 bootstrap replicates. The result showed that LcSVP2 clustered with AT2G22540 (SVP/AGL22) and AT2G24540 (SVP/AGL24) from Arabidopsis; Os02g52340, Os06g11330, and Os03g08754 from *O. sativa*; and the six DAMs from *P. persica* (Fig. 1b).

### Expression pattern of *LcSVP2* in different tissues of litchi

**Figure 2:**
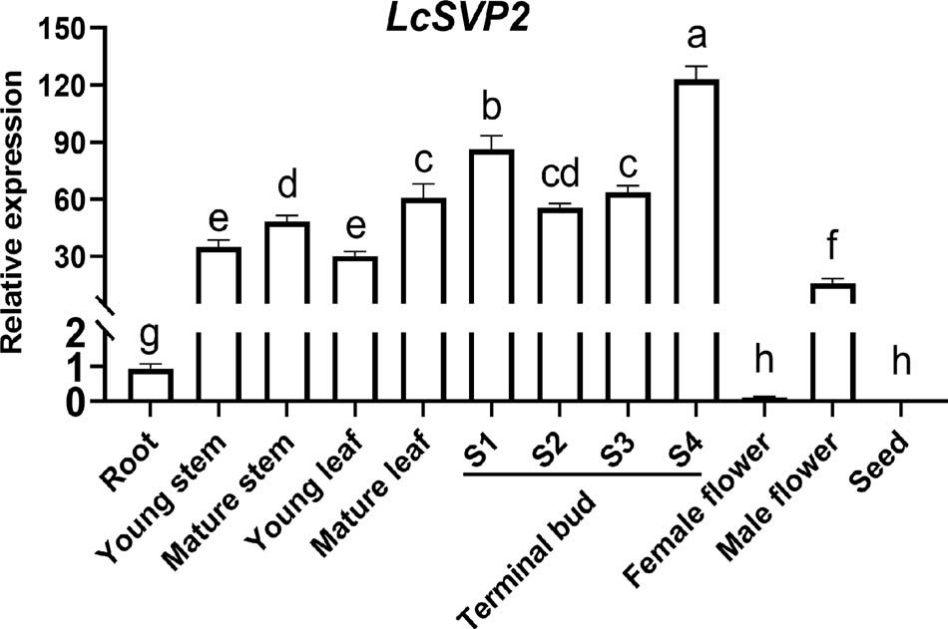
Expression profile of *LcSVP2* in different tissues of litchi. Error bars represent standard error of means (*n* = 3). Different letters indicate significant differences at *P* < .05, according to Tukey’s multiple range test (*n* = 3)

To explore the molecular function of *LcSVP2*, we first analyzed the expression profile of *LcSVP2* in different tissues by qRT-PCR. The transcript levels of *LcSVP2* were relatively high in the terminal buds, stems, leaves, and male flowers, but low in the roots, and almost undetectable in the female flowers and seeds (Fig. 2). Highest expression of *LcSVP2* was found in the terminal buds at growth cessation stage (Stage 4) and at dormant stage (Stage 1). The transcript level of *LcSVP2* significantly decreased after dormancy removal in Stage 2 and Stage 3 but dramatically increased in Stage 4 (growth cessation stage; Fig. 2), which agreed with the result of Zhang et al. [4]. The results indicate that *LcSVP2* may have a close association with dormancy onset in the terminal bud of litchi.

### Overexpression of *LcSVP2* in Arabidopsis delays flowering

**Figure 3:**
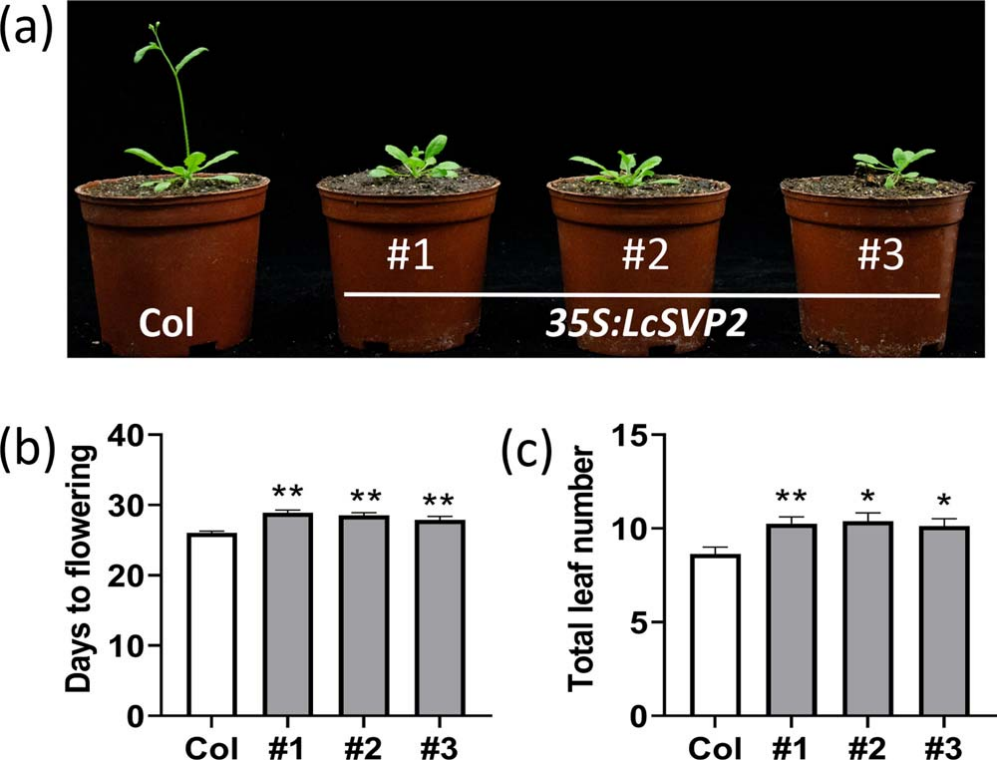
Functional analysis of *LcSVP2* in Arabidopsis. (a) Phenotype of *35S:LcSVP2* transgenic and WT Arabidopsis plants. (b) Days to flowering and (c) numbers of leaves to flowering of *35S:LcSVP2* transgenic and WT plants under long day. The number of leaves and flowering time were counted when plants bolted to about 1 cm. Error bars represent standard error of means; * and ** indicate significant difference between the *35S:LcSVP2* transgenic plants and WT plants at *P* < .05 and *P* < .01, respectively, Student’s *t*-test

To further investigate the role of *LcSVP2* in plant development, we isolated it from litchi shoot bud (Fig. S1), and transformed it into the model plant Arabidopsis with the constitutive *35S* promoter. A total of eight transgenic plant lines of *35S:LcSVP2* were obtained and three independent transgenic lines were randomly selected in the T4 generation (Fig. S2) for phenotypic analysis. The number of leaves and flowering time when plants bolted to about 1 cm were recorded. Compared with the wild type, all the T4 transgenic plants exhibited a late flowering phenotype (Fig. 3a) in terms of both days to flowering (Fig. 3b) and the total leaf number (Fig. 3c), indicating that *LcSVP2* acted as a flowering repressor.

### Silencing *LcSVP2* delays re-entry of dormancy in litchi buds

**Figure 4:**
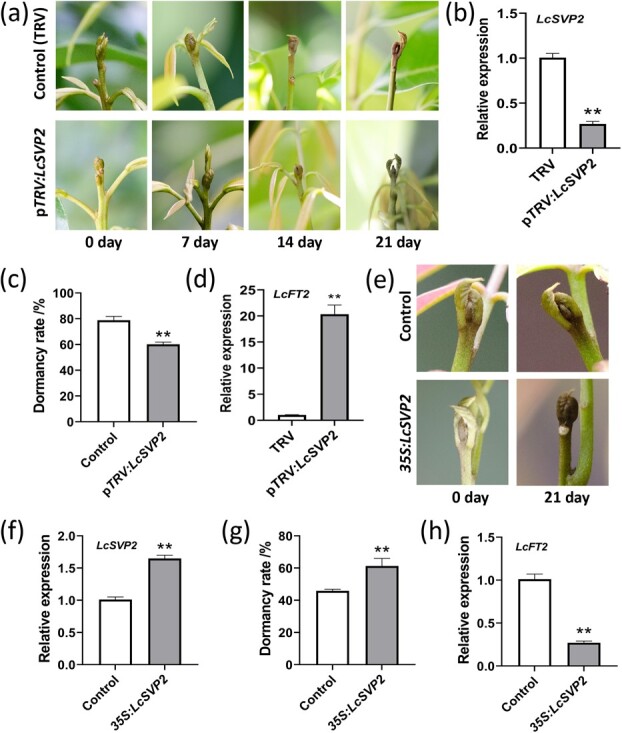
Functional analysis of *LcSVP2* in litchi terminal bud. (a) Morphology of terminal buds in *LcSVP2* silenced group and the control. (b) The expression level of *LcSVP2* in p*TRV:LcSVP2* and control buds. (c) Effect of silencing *LcSVP2* on budbreak. (d) The expression levels of *LcFT2* in p*TRV:LcSVP2* and control buds. (e) Morphology of *35S:LcSVP2* terminal buds and the control buds. (f) The expression level of *LcSVP2* in *35S:LcSVP2* and control buds. (g) Effect of overexpressing *LcSVP2* on budbreak. (h) The expression levels of *LcFT2* in *35S:LcSVP2* and control buds. Error bars represent standard error of means; * and ** indicate significant difference between the *35S:LcSVP2* transgenic plants and WT plants at *P* < .05 and *P* < .01, respectively, Student’s *t*-test

To further confirm the function of *LcSVP2* in dormancy development of litchi terminal bud, a VIGS assay was employed to silence *LcSVP2* in litchi terminal bud at early Stage 3. The terminal buds in p*TRV:LcSVP2* group entered dormancy later than in the control group treated with empty p*TRV* (Fig. 4a), and *LcSVP2* expression in p*TRV:LcSVP2* group was significantly reduced (Fig. 4b), resulting in significantly lower dormancy rate compared to the control (Fig. 4c). The result confirms that *LcSVP2* is essential to the onset of bud dormancy. The expression of the flowering related gene, *LcFT2*, was significantly increased when *LcSVP2* was silenced (Fig. 4d).

Furthermore, overexpression transformation of *LcSVP2* via *35S* promoter in terminal buds was also performed. The terminal buds at early Stage 3 were used for transient overexpression (Fig. 4e). Twenty-one days after the treatment, the expression of *LcSVP2* and the average dormancy rate significantly increased in *35S:LcSVP2* treated terminal buds (Fig. 4f and g). The expression of *LcFT2* was significantly reduced when *LcSVP2* was overexpressed (Fig. 4h). These results further indicate that *LcSVP2* plays a role in the dormancy entry of litchi terminal buds and inhibits the expression of *LcFT2* gene.

### LcSVP2 is located in the nucleus and has transcriptional inhibitory activity

**Figure 5:**
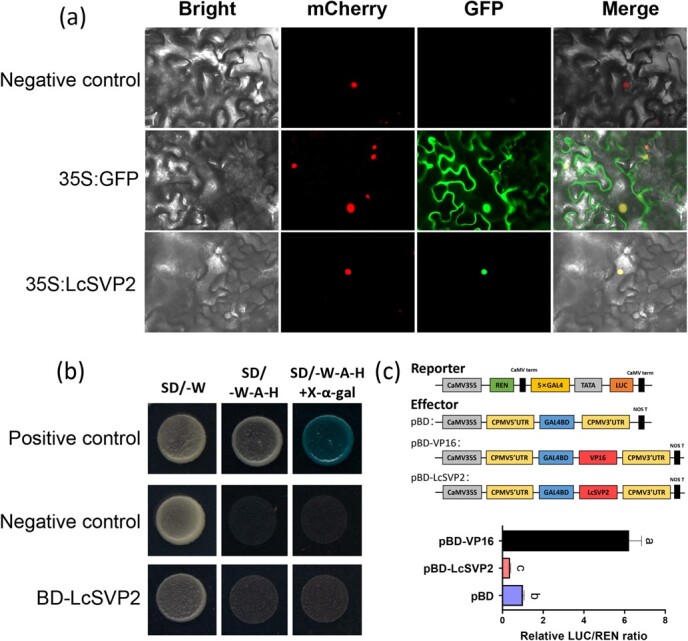
Subcellular localization and transcriptional activity analysis of LcSVP2. (a) Subcellular location of LcSVP2 in *N. benthamiana* leaf cells. Green color represents the fluorescence of GFP and red color represents the fluorescence of nuclear marker VirD2NLS-mCherry. (b) Transactivation analysis of the LcSVP2 in yeast. pGBKT7–53 + pGADT7-T group was used as the positive controls, pGBKT7-Lam group as the negative controls. (c) Transcriptional repression assay of LcSVP2 in tobacco leaves. Different colors represent different functional regions on the reporter and effector. Six biological replicates were performed for each value, and vertical bars represent standard deviation. Error bars represent standard error of means. Different letters indicate significant differences at *P* < .05, according to Tukey’s multiple range test

To better understand the molecular function of LcSVP2 protein, subcellular localization was performed with VirD2NLS–mCherry used as the nucleus marker. In the 35S:GFP group, the fluorescence was showed throughout the cell, while the fluorescence was showed just in the nucleus in the 35S:LcSVP2-GFP group, suggesting that LcSVP2 is a nuclear protein (Fig. 5a).

To explore the transcriptional activity of LcSVP2, the CDS of *LcSVP2* was fused in the pGBKT7 vector (BD), and then transformed into the yeast strain AH109 (Fig. 5b). All of the yeast cells established normally on SD/−Trp medium. However, only the positive control yeast cells transformed with pGBKT7−p53 survived when grown in the selective medium SD/−Trp/−His/−Ade. When grown on selective medium SD/−Trp/−His/−Ade, neither yeast cell transformed with BD−LcSVP2 nor the empty vector pGBKT7 survived. To further confirm whether LcSVP2 is a transcriptional inhibitor, a LUC reporter assay was designed (Fig. 5c). The result showed that LcSVP2 significantly inhibited LUC activity compared with the control pBD (Fig. 5c), indicating that LcSVP2 primarily functions as a transcriptional inhibitor.

### LcSVP2 interacts with LcSMOS1

**Figure 6:**
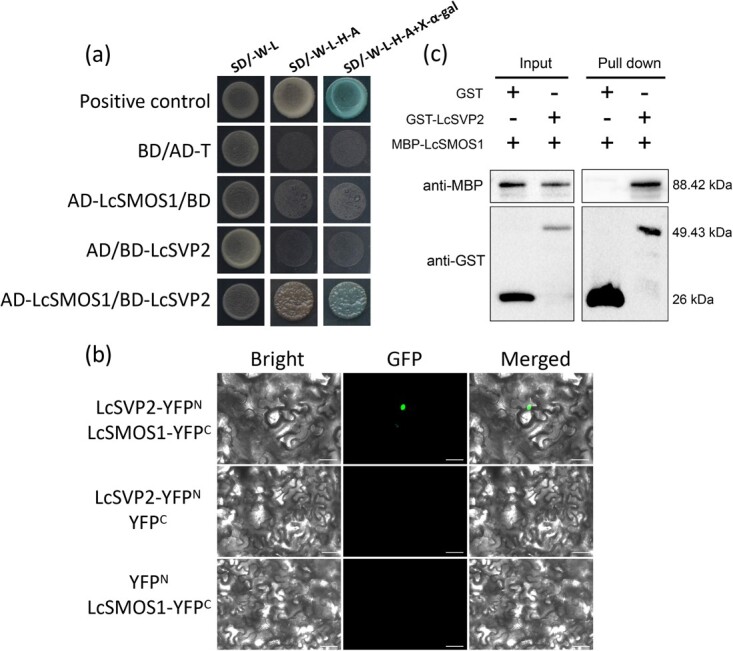
Interaction between LcSVP2 and LcSMOS1. (a) Y2H assays showing interactions between LcSVP2 and LcSMOS1. pGBKT7–53 + pGADT7-T was used as the positive controls. (b) Glutathione-S-transferase (GST) pull-down assays of the interaction between LcSVP2 and LcSMOS1. Bands were detected using an anti-GST antibody. (c) The BiFC assay in tobacco leaves shows the interaction between LcSVP2 and LcSMOS1

To determine how LcSVP2 is involved in the regulation of terminal bud endodormancy and to explore the proteins that may interact with LcSVP2 in litchi, the Y2H library was created from litchi shoot buds collected. The AP2-type transcription factor LcSMOS1 (LITCHI008837) protein was screened that may interact with LcSVP2. To further confirm the interaction between LcSVP2 and LcSMOS1, pGADT7−LcSMOS1 was used as prey for Y2H assay along with pGBKT7−LcSVP2 (Fig. 6a). The result indicates that LcSVP2 was able to interact with the LcSMOS1 (Fig. 6a). The bimolecular fluorescence complementation (BiFC) assay result was consistent with the result of Y2H (Fig. 6b). Moreover, the pull-down assay also confirmed the interaction between LcSVP2 and LcSMOS1 (Fig. 6c). The results proved the interaction between LcSVP2 and LcSMOS1.

### LcSMOS1 is essential for dormancy maintenance

**Figure 7:**
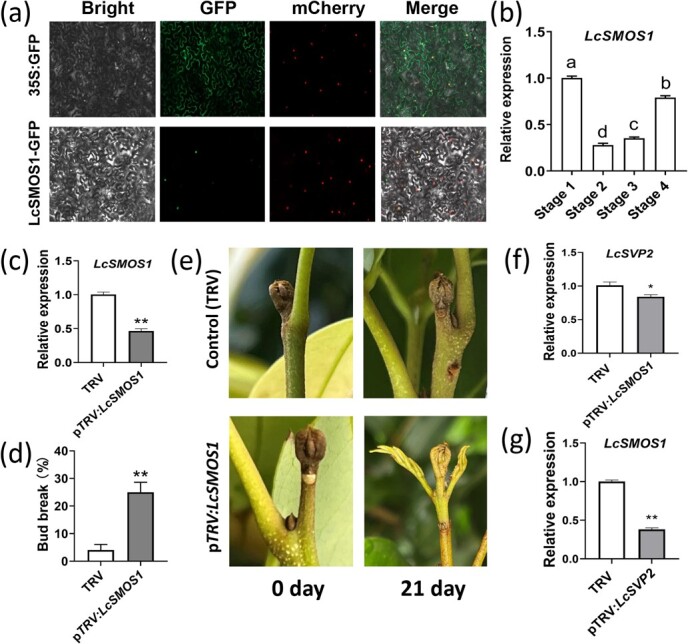
The functional characteristics of LcSMOS1. (a) Subcellular localization of LcSMOS1. Green color represents the fluorescence of GFP and red color represents the fluorescence of nuclear marker VirD2NLS-mCherry. (b) The expression of *LcSMOS1* in terminal bud of litchi at different stages. (c) The expression of *LcSMOS1* in p*TRV:LcSMOS1* and control buds. (d) Effect of silencing *LcSMOS1* on budbreak and (e) bud morphology. (f) The expression of *LcSVP2* in p*TRV:LcSMOS1* and control buds. (g) The expression of *LcSMOS1* in p*TRV:LcSVP2* and control buds. Error bars represent standard error of means (*n* = 3). Different letters indicate significant difference among stages at *P* < .05, Tukey’s multiple range test (*n* = 3); * and ** represent significant difference at *P* < .05 and *P* < .01, Student’s *t*-test (*n* = 3)

To better understand the characteristics of the LcSMOS1 protein, the conservative structural domain was analyzed using NCBI Conserved Domain Database, and a typical AP2 domain was found in LcSMOS1 (Fig. S3a). Sequence alignment analysis of the litchi *LcSMOS1* and Arabidopsis *AtSMOS1* (AT2G41710) revealed the conserved AP2 domain and high sequence identity (66%) (Fig. S3b). In addition, subcellular localization assay suggested that LcSMOS1 localized in the nucleus (Fig. 7a).

The expression profile of *LcSMOS1* in the terminal buds at different stages showed that *LcSMOS1* was highly expressed in the Stage 1, significantly decreased in Stage 2 and Stage 3, and then increased in Stage 4 (Fig. 7b). The expression trend of *LcSMOS1* was consistent with that of *LcSVP2*.

To further perform the functional characterization of *LcSMOS1*, a VIGS assay was used to suppress *LcSMOS1* in litchi terminal buds. Interestingly, 21 days after VIGS treatment, *LcSMOS1* expression was significantly reduced (Fig. 7c); the average budbreak rate was significantly increased (Fig. 7d); and the outgrowth of the terminal buds in the p*TRV:LcSMOS1* treatment was clearly advanced (Fig. 7e). These results suggested that, similar to LcSVP2, LcSMOS1 may also act as an essential regulator in maintenance of dormancy in litchi terminal buds. Interestingly, the *LcSVP2* expression was greatly down-regulated in the p*TRV:LcSMOS1* plants (Fig. 7f), as was *LcSMOS1* expression in p*TRV:LcSVP2* plants (Fig. 7G). These findings suggest that the expression of *LcSVP2* and *LcSMOS1* affects each other and jointly participate in the regulation of terminal buds dormancy in litchi.

### Ethylene is low in growing terminal buds and ethephon enforces bud dormancy

**Figure 8:**
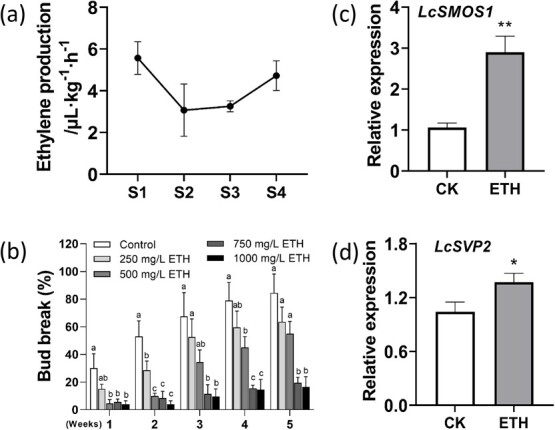
Changes in ethylene evolution rate in terminal buds at different stages and effects of ethephon treatment on budbreak and relative expression of *LcSVP2* and *LcSMOS1* in terminal buds. (a) Changes in ethylene production in terminal buds at different stages. (b) The effect of different concentrations of ethephon on budbreak. (c) Relative expression of *LcSVP2* and (d) *LcSMOS1* in litchi terminal buds 7 days after treatment with 1000 mg/L ethephon. Error bars represent standard error of means (*n* = 4). Different letters indicate significant difference among stages at *P* < .05, Tukey’s multiple range test; ^*^ and ^**^ represent significant difference at *P* < .05 and *P* < .01, Student’s *t*-test (*n* = 4)

Considering that LcSMOS1 is a member of the AP2/ERF big family, and ethylene plays a crucial role in the bud dormancy [30]. Ethylene production rate was measured in terminal buds at different stages (Fig. 8a). Ethylene evolution rate from Stages 1 terminal buds was the highest (5.6 μL・kg^−1^・h^−1^); whereas that at Stages 2 and Stages 3 was 3.1 μL・kg^−1^・h^−1^ and 3.2 μL・kg^−1^・h^−1^, respectively. However, ethylene evolution rate from buds at growth cessation (Stage 4) increased to 4.7 μL・kg^−1^・h^−1^ (Fig. 8a). These results show that the endogenous ethylene levels in dormant terminal buds stays high. In addition, we used different concentrations of ethylene releaser (ethephon) to treat litchi terminal buds and found that ethephon effectively inhibited budbreak, the effect being stronger at higher concentrations (Fig. 8b). The expressions of *LcSMOS1* and *LcSVP2* were also significantly up-regulated by ethephon treatment (Fig. 8c and d). The results indicate that *LcSMOS1* and *LcSVP2* are involved in dormancy maintenance of litchi terminal buds in response to ethylene.

### ABA is high in dormant terminal buds and effective to enforce bud dormancy

**Figure 9:**
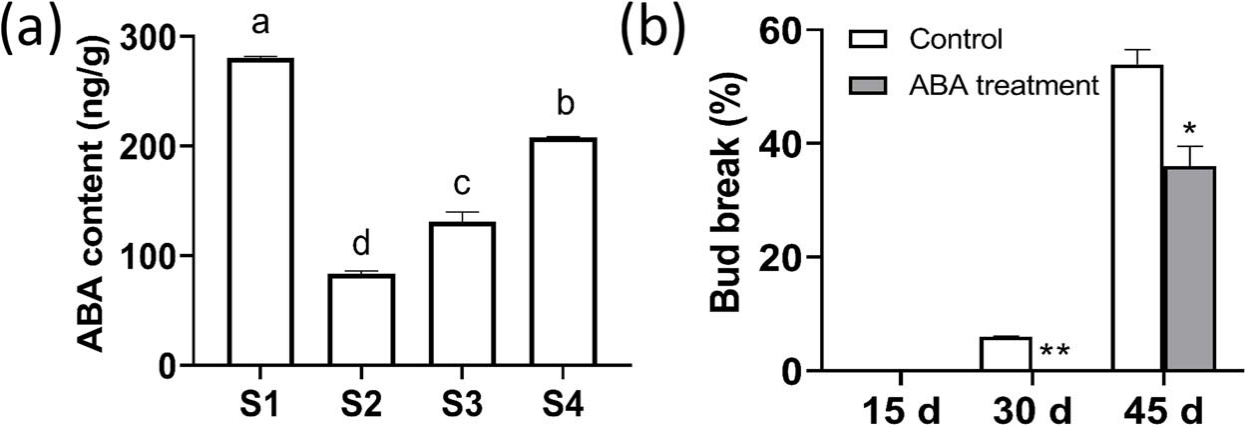
Changes in ABA content in terminal buds at different stages (a) and effect of exogenous ABA (200 mg/L) treatment on budbreak (b). Error bars represent standard error of means (*n* = 3). Different letters indicate significant difference among stages at *P* < .05, Tukey’s multiple range test (*n* = 3); ^*^ and ^**^ represent significant difference at *P* < .05 and *P* < .01, Student’s *t*-test

ABA is one of the most important hormones which plays a crucial role in plant dormancy [12, 26]. ABA content in litchi terminal buds at different stages was detected. Endogenous ABA was the highest in dormant buds at Stage 1, and decreased sharply in buds at Stage 2, while increased constantly toward Stage 4 (Fig. 9a). Exogenous ABA treatment at 200 mg/L significantly inhibited and delayed budbreak (Fig. 9b, Fig. S4). These findings suggest that ABA plays a key role in maintaining the dormancy of litchi terminal buds.

### ABA-induced a short-term up-regulation of *LcSVP2* and *LcSMOS1*, and *LcSVP2* promoted *LcNCED3* expression

**Figure 10:**
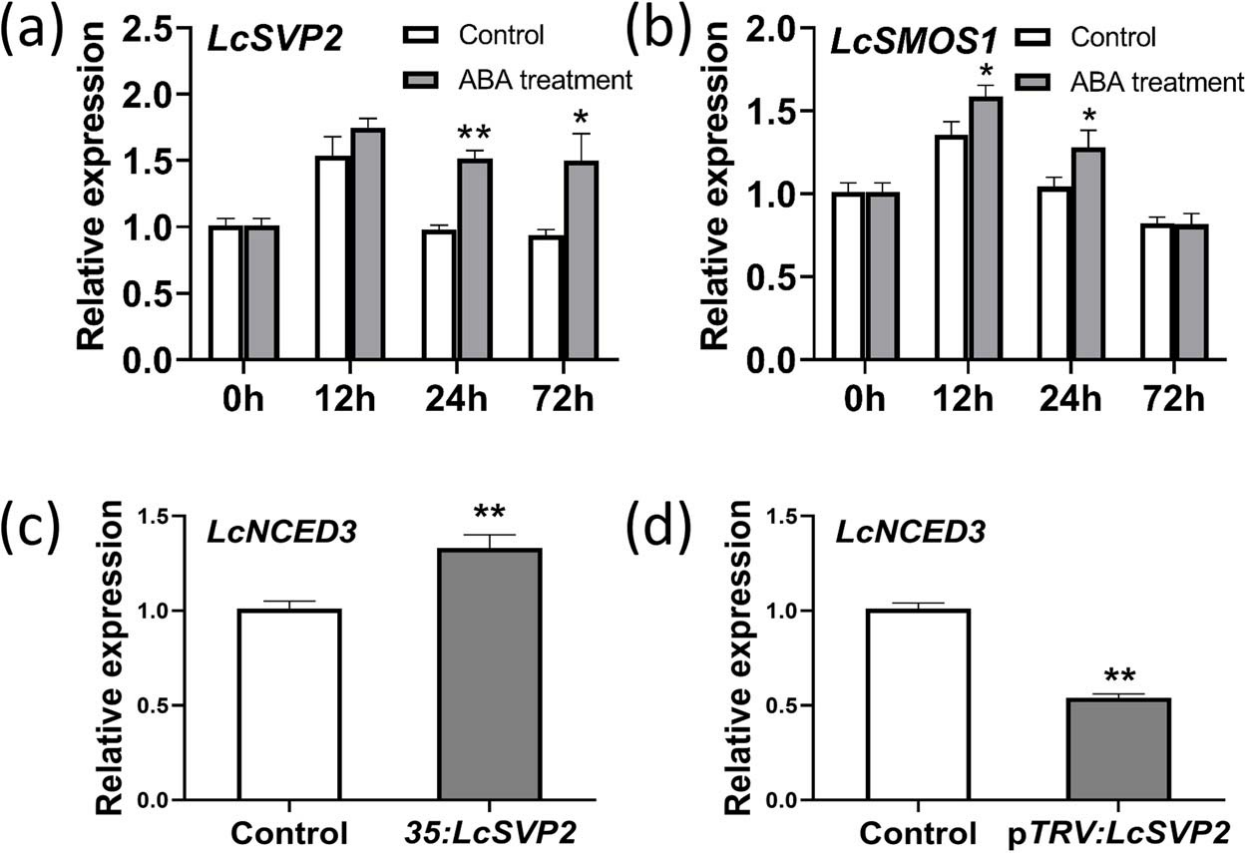
Effects of ABA treatment at Stage I on the expression of *LcSVP2* (a) and *LcSMOS1* (b). Vertical bars represent standard errors of means (*n* = 3); ^*^ and ^**^ indicate significant differences at *P* < .05 and *P* < .01, Student’s *t*-test, respectively

To further investigate whether the expressions of *LcSVP2* and *LcSMOS1* were responsive to ABA, we conducted ABA treatments and qRT-PCR assays. Under ABA treatment, the expression of *LcSVP2* in the terminal buds significantly increased within 72 h after the treatment (Fig. 10a). The expression of *LcSMOS1* was also significantly enhanced within 24 h after ABA treatment (Fig. 10b). The results suggest that ABA-induced a short-term increase in *LcSVP2* and *LcSMOS1* expression in the terminal buds of litchi.

On the contrary, considering that DAM/SVPs can upregulate ABA synthesis in previous studies from other species [19, 20], we further checked the effect of *LcSVP2* on ABA synthesis. The expression of *LcNCED3*, an ABA biosynthetic gene, was detected in the terminal buds treated with *35S:LcSVP2* and p*TRV:LcSVP2* and in control buds. The expression of *LcNCED3* in the terminal buds was also significantly enhanced and decreased by *35S:LcSVP2* (Fig. 10c) and p*TRV:LcSVP2* (Fig. 10d), respectively, indicating that *LcSVP2* may promote the synthesis of ABA.

## Discussion

Dormancy of the terminal bud in evergreen litchi, enables interruption of outgrowth of tender leaves so that pests infesting tender leaves have not continuous food supply, which is a smart strategy to suppress pest outbreak [4]. Timely control of terminal bud dormancy is crucial for litchi flowering as outgrowth of vegetative flush in winter minimizes winter chill induced flowering [31]. The understanding of molecular mechanisms of dormancy regulation in litchi will provide important theoretical references for precise control of flush cycle in litchi and other evergreen perennials. The MADS-box transcription factors, SVPs, have proved to suppress flowering in the model plant Arabidopsis [32–34]. However, in temperate deciduous fruit trees, SVP-like MADS-box transcription factors were found to be associated with bud dormancy, and therefore they are called ‘*DAM*’ genes [8]. Our previous study suggested a *SVP-like* gene *LcSVP2* might be responsible for dormancy onset and maintenance in terminal bud of litchi [4]. However, the role of *LcSVP2* in litchi bud dormancy was not well illustrated. In this study, we provided some evidences on the function of *LcSVP2* in regulating dormancy of litchi terminal buds and its response to hormones.

### *LcSVP2* plays dual roles in regulating dormancy onset and flowering in litchi

In Arabidopsis, the expression of *AtSVP* has been reported to be high in young leaves and shoot meristem, but low in flowers and siliques [15]. According to a previous study in the perennial fruit tree of trifoliate orange, a high expression level of *PtSVP* was shown in the shoot, indicating that *PtSVP* may be closely related to shoot development [35]. However, in this study, high expression of *LcSVP2* was observed in the terminal buds, stems, leaves, and male flowers. High expression was found in terminal buds at growth cessation and dormancy stages but much lower levels in root, female flower, and seed. This result agrees with our previous study [4], indicating that *LcSVP2* may chiefly be involved in the regulation of shoot growth instead of root growth and has a role in regulating entrance and maintenance of dormancy in litchi terminal buds. The sharp difference in *LcSVP2* expression between male and female flowers suggests a likely role of this gene in sex determination, which requires further investigation.

In this study, silencing *LcSVP2* in terminal buds at early Stage 3 delayed the onset of dormancy, which clearly showed that *LcSVP2* is a key regulator gene responsible for dormancy onset in litchi terminal bud. Therefore, LcSVP2 shares the function features in regulating bud dormancy with the DAMs identified in temperate deciduous trees [8].

The SVP proteins from different species often serve varied roles in plant development, including flowering development, floral meristem development, pedicel elongation development, lateral inflorescences development, and others [15, 36–38]. As abovementioned, SVPs inhibit flowering in the model plant of Arabidopsis [32–34]. The *svp* Arabidopsis mutant plant caused early flowering, and overexpression in plant delayed flowering [15]. Ectopic expression of some of *DAMs* or *SVPs* from temperate plants in Arabidopsis induced flower abnormalities or delayed flowering, indicating that they also function as a flower repressor [39, 40]. Studies have proven that the number of cauline branches is closely related to flowering time in Arabidopsis [41, 42]. And Flowering Locus T (FT) also regulates floral transition of the axillary meristem by interacts with BRANCHED1, then participates in branching pattern regulation [43]. However, in *35S:LcVSP2* Arabidopsis, flowering was delayed but no significant change in branching was observed. Interestingly, DAMs or SVPs may regulate dormancy or flowering through suppressing *FT* transcription ([17, 18], Falavigna et al. 2018). In litchi, expression levels of the three *LcSVPs* decease in response to chilling exposure, which induces *LcFT1* and flowering [24], while it was increased by brassinosteroid treatment that reduced *LcFT1* expression and flowering, suggesting the LcSVPs might act as a flowering suppressor in litchi [29]. In the current study, ectopic expression of *LcSVP2* significantly delayed flowering in Arabidopsis. Silencing *LcSVP2* in litchi terminal up-regulated *LcFT2*, another *LcFT*, whose overexpression triggered flowering in Arabidopsis and tobacco [24]. Therefore, *LcSVP2* plays dual roles in promoting dormancy onset and suppressing flowering in litchi. This explains why dormant litchi buds with a high *LcSVP2* transcript level fail to respond to chilling temperatures and produce flower [44].

### LcSVP2 interacts with LcSMOS1, both crucial for dormancy

The present study showed LcSVP2 interacted with an AP2-type transcription factor LcSMOS1. AP2 transcription factor family is discovered mainly in plants, and characterized by AP2/ERF domain [45]. The AP2/ERF domain was first identified in AP2 transcription factor of Arabidopsis [46]. In Arabidopsis studies, AP2 plays a key role in the regulation of flower and shoot apical meristem development [47, 48]. AtSMOS1 was reported to have function of regulation cell size in Arabidopsis [49], it always performs functions in cooperation other floral meristem genes. For example, *AtSMOS1* and *APETALA1* (*AP1*), *LEAFY* (*LFY*) genes have a close relationship in terms of functional performance [47]. Up till now, hundreds of AP2/ERFs have been discovered and identified from various plants, including perennial woody fruit trees, e.g. grapevine [50], apple [51], longan [52], and peach [53]. However, the roles of SMOS1 in woody plants still remain largely unknown. In this study, *LcSMOS1* was found to be highly expressed in dormant buds and buds entering dormancy, similar to *LcSVP2*. LcSMOS1 proved to be crucial for dormancy maintenance as silencing *LcSMOS1* advanced outgrowth of the terminal bud. Therefore, both LcSVP2 and LcSMOS1 are involved in dormancy regulation. Interestingly, expression of the two genes is closely associated as silencing one of them lowered the expression of the other. However, the biological significance of the LcSVP2–LcSMOS1 complex remains further exploration.

### *LcSVP2* and *LcSMOS1* respond similarly to dormancy enforcing hormones

It is well established that onset and maintenance of bud dormancy in temperature perennials is mediated by endogenous ABA, which increases under short-day and chilling conditions that induces dormancy [12, 26, 27]. DAM–SVPs are considered as downstream transcript factors in ABA-involved regulation pathway of endodormancy establishment [5, 54]. Tuan et al. [19] found ABA biothesis gene *PpNCED3* was activated by PpDAM1 in pear, which led to increased endogenous ABA during endodormancy, while PpAREB1 (ABA response element-binding) transcription factor, negatively regulated *PpDAM1*, leading to release of endodormancy. In the present study, endogenous ABA in litchi terminal bud was highest at dormant stage (Stage 1), decreased during budbreak and increased again towards growth cessation (Stage 4). Exogenous ABA induced significant increases in both *LcSVP2* and *LcSMOS1* within 72 h after treatment and delayed budbreak of litchi*.* This result again shows the close transcription association of the two genes and indicates that ABA might enforce litchi bud dormancy by up-regulating *LcSVP2* and *LcSMOS1*.

Ethylene has been shown to play a positive role in seed dormancy release [55]. In deciduous grape vine, budbreak can be promoted by exogenous gaseous ethylene [56, 57], and ethylene signaling plays a crucial role in bud dormancy release [30]. However, our findings and those obtained by Cronje et al. [28] demonstrated that exogenous ethylene suppressed budbreak in litchi. The ethephon treatment up-regulated *LcSVP2* in litchi terminal buds [28]. Our study showed that ethylene release rate from terminal bud was highest during dormant stage, and reduced in breaking and fast growth stages and increased again during growth cessation stage. Additionally, our study also showed treatment with ethephon promoted the expression of *LcSVP2* and *LcSMOS1* in terminal buds of litchi.

## Conclusion

*LcSVP2* in litchi terminal bud is highly expressed during entry of dormancy and silencing this gene postponed dormancy re-entry of the growing terminal buds, while overexpressing *LcSVP2* in terminal buds inhibits budbreak, proving this gene plays a vital role in dormancy onset in evergreen tree of litchi. Ectopic expression of *LcSVP2* in Arabidopsis results in delayed flowering, suggesting a dual role of LcSVP2 in regulating flowering as well as dormancy. LcSVP2 interacts with LcSMOS1, and silencing of *LcSMOS1* promotes budbreak, suggesting that LcSMOS1 also plays an essential role in dormancy regulation. Ethylene and ABA enforce dormancy of litchi bud possibly through up-regulating *LcSVP2* and *LcSMOS1*, transcription of which shows close association. The study provides new insights into the molecular mechanisms of bud dormancy regulation in tropical evergreen fruit trees.

## Materials and methods

### Plant materials

The experiment was performed in South China Agricultural University, Guangzhou, Guangdong, China. A 20-year-old ‘Feizixiao’ litchi was used as the material in this study. The terminal buds in different developmental stages, i.e. dormant stage (Stage 1), budbreak stage (Stage 2), rapid growth stage (Stage 3), and growth cessation stage (Stage 4) were collected according to our previous report [4]. Root, young and mature stems, young and mature leaves, and female and male flowers were collected on 20 March 2022, during the blooming of litchi. Fully developed seeds were collected on 16 May 2022. Terminal buds were sampled from adult litchi trees of ‘Feizixiao’. Three tree-based biological replicates per stage or organ were used.

The Columbia wild-type (WT) Arabidopsis (*A. thaliana*) was used in this study. The seeds of Arabidopsis were first sterilized with 75% ethanol and 10% sodium hypochlorite, then vernalized at 4°C for 4 days on the medium of 1/2 Murashige and Skoog. 10 days-old seedlings were grown into soil in a greenhouse at 23°C under a long day (16/8 h, light/dark) conditions.

### Hormone treatments

For ABA treatment assays, three ‘Feizixiao’ trees with terminal buds at Stage 1 were selected on 21 August 2023. The canopy of each tree was vertically divided into two even zones, which were randomly assigned to spray of ABA solution at 200 mg/L (756.7 μM; containing 0.05% Tween-20), and to spray of clean water containing 0.05% Tween-20 as the control. The terminal buds were sprayed until drip-off. Bud samples were taken at 0, 12, 24, and 72 h after treatment. Terminal buds with outgrowth of new shoots from randomly selected 50 terminals in each treatment of each tree were recorded at intervals of 15 days and budbreak rate was calculated for each recording time.

For ethephon treatment, twenty 20-year-old litchi trees of ‘Feizixiao’ with terminal flush fully mature before outgrowth of new flush were selected on 1 November 2023. They were randomly divided into five groups and assigned to spray with ethephon solutions at 0 mg/L (control), 250 mg/L (1.7 mM), 500 mg/L (3.5 mM), 750 mg/L (5.2 mM), and 1000 mg/L (6.9 mM) diluted in 0.05% Tween-20, respectively. The control group was sprayed with a clean water containing 0.05% Tween-20 only. Seven days after treatment with 1000 mg/L ethephon, about 50 terminal buds were collected from each tree and the samples were immediately frozen using liquid nitrogen for ribonucleic acid (RNA) extraction and quantitative reverse transcription polymerase chain reaction (qRT-PCR) assays. Terminal bud status of randomly selected 50 shoot terminals from each tree was recorded weekly after treatment.

### Ribonucleic acid extraction and quantitative reverse transcription polymerase chain reaction

Total RNA of the litchi terminal bud sample was extracted using the RNeasy Plant Mini Kit (Huayueyang, Beijing, China). The first-strand copy deoxyribonucleic acid (cDNA) was generated using SMARTScribe Reverse Transcriptase Kit (Takara, Shanghai, China) according to the instructions. qRT-PCR reaction was performed using ABI QuanStudioTM 12K Flex (Thermo Fisher, CA, USA). Three tree-based biological replicates, each with three technical replicates were performed. The expression dates were analyzed using the 2^-△△CT^ method [58]. *LcActin* was used as the internal reference gene. The primers used are listed in Table S1.

### Isolation of the *LcSVP2* and *LcSMOS1*

To obtain the CDSs of *LcSVP2* and *LcSMOS1*, the PCR program was performed using their specific primers designed by Primer 5, which were listed in Table S1. The reference sequences of *LcSVP2* and *LcSMOS1* for primers design were obtained from the database at http://www.sapindaceae.com/ [25]. The PCR protocol consisted of 36 cycles of 3 min at 95°C, 15 s at 95°C, 15 s at 58°C, and 30 s at 72°C, followed by 10 min at 72°C.

### Phylogenetic analysis of LcSVP2

Sequence alignment of the LcSVP2 and homologous proteins (AGL22/24) of SVP2 selected from Arabidopsis was performed using ClustalX. For phylogenetic analysis, LcSVP2 and 76 members of MIKC type MADS-box family members from Arabidopsis and 61 members of *Oryza sativa* obtained from Plant Transcription Factor Database (https://planttfdb.gao-lab.org/) were analyzed alongside 6 DAM proteins (NCBI accession number, DAM1 to DAM6: DQ863253, DQ863255, DQ863256, DQ863250, DQ863251, and DQ863252) of *Prunus persica* obtained from NCBI Database. The analysis was performed using MEGA version 7.0 by 1000 bootstrap replicates with the Neighbor-Joining (NJ) method [59, 60].

### Vectors construct and plant transformation

To construct the vectors, the pMD19-T vector was used. The PCR results underwent digestion with XbaI and KpnI, and subcloned into the overexpression vector Super1300-cGFP. Then, the Super1300-cGFP vector with LcSVP2 and LcSMOS1 plasmid was introduced into *Agrobacterium tumefaciens* strain GV3101. The floral dip approach was employed for Arabidopsis plant transformation, following a prior publication [61]. Transgenic plants were further selected on the MS medium with 50 mg L^−1^ hygromycin B. Three homozygous lines of the T4 generations were used for further phenotype observation.

### Subcellular localization assay

Subcellular localization assay of LcSVP2 and LcSMOS1 were performed according to a previous report [62]. The coding sequences of *LcSVP2* and *LcSMOS1* without the stop codon were inserted into the Super1300-cGFP vector. The fusion vectors with GFP were then converted into *A. tumefaciens* GV3101, and the Agrobacterium liquid was infiltrated into *N. benthamiana* leaves. 3 days later, the infected leaves were observed under a Confocal Re-Imagined microscopy. VirD2NLS-mCherry [63] was used as a nuclear marker.

### Transcriptional activation assays

Transcriptional activation of LcSVP2 was performed with yeast according to [64]. The CDS of *LcSVP2* was inserted into the pGBKT7 vector. Subsequently, the pGBKT7–LcSVP2 fusion vector was transformed into the strain AH109 yeast cell by the previous described method [65]. The yeast cells were plated on synthetic dropout (SD) medium without tryptophane (SD/−Trp), and then put into an incubator and cultured at 30°C for 72 h. The positive clones were selected on SD medium without tryptophane, histidine, and adenine (SD/−Trp−His−Ade), and cultured at 30°C for 72 h. The pGBKT7 was used as the negative control.

For dual-luciferase (LUC) reporter assay, the CDS of *LcSVP2* was constructed into the pBD vector as an effector. The GAL4-binding elements and LUC reporter gene driven by the *35S* promoter were used as a reporter. The effector and reporter vectors were then combined into *A. tumefaciens* GV3101 and infiltrated into the healthy leaves of *N. benthamiana*. 3 days later, the activity ratio of the LUC/REN was detected according to a previous study [66]. The primers used in this experiment are provided in Table S1.

### Construction of cDNA yeast two-hybrid library of litchi terminal buds

To explore the regulatory pathways of LcSVP2 protein, a yeast two-hybrid (Y2H) cDNA library of litchi terminal buds was constructed and the proteins that interact with the LcSVP2 were screened. The library’s construction was carried out in accordance with a previous study [67]. Fresh terminal buds of ‘Feizixiao’ were collected and froze in liquid nitrogen. Total RNA was extracted and double-strand cDNAs was synthesized using the cDNA Synthesis Kit (Invitrogen, Shanghai, China). The cDNAs were ligated to the pGADT7 vector to generate library recombinant vectors, which were subsequently electroporated into competent *Escherichia coli* (*E. coli*) TOP10 cells. For Y2H library screening, the method was used as previously described [68], and the library plasmid was extracted using a plasmid mini preparation kit (Beyotime Biotechnology, Shanghai, China).

### Yeast two-hybrid analysis

For yeast Y2H assay, the CDS of *LcSVP2* was amplified using primers containing the EcoRI and BamHI sites and inserted into the pGBKT7 vector used as the bait (BD-LcSVP2). Full-length *LcSMOS1* CDS amplified by primers containing EcoRI and XhoI sites and inserted into pGADT7 used as the prey (AD-LcSMOS1). The analyses of protein interactions were performed in accordance with a previous report [64]. Briefly, yeast cells were incubated at 30°C condition for 72 h on SD medium without Leu and Trp, then grown in SD medium without His, Ade, Leu, and Trp which containing X-α-gal to determine binding activity.

### Bimolecular fluorescence complementation assay

The CDSs of *LcSVP2* and *LcSMOS1* were subcloned into the vector pSPYNE-35S and pSPYCE-35S, respectively. The construct vectors were transformed into *A. tumefaciens* strain GV3101 and used for infiltration test which was performed as the method described by [64]. Briefly, equal volumes of agrobacterium suspension were mixed with MES buffer (10 mM MES, 10 mM MgCl_2_, 150 μM acetosyringone, pH 5.6) and injected into *N. benthamiana* tobacco leaves. The transfected tobacco plants were grown in the greenhouse for 3 days at 22°C condition. The result of LcSVP2 and LcSMOS1 interaction was observed using confocal microscopy. The primers used are provided in Table S1.

### Pull-down assay

A protein interaction assay pull-down experiment was performed according to a previous study [64]. pGEX-4T-1 and pMal-c2x vectors were used to construct GST-tagged recombinant protein and MBP-tagged recombinant protein, respectively. GST and MBP fusion proteins were expressed in *E. coli BL21* strain and bound to GST and MBP bind resin (Yeasen Biotechnology), respectively. The GST and fusion proteins GST-LcSVP2/MBP-LcSMOS1 were incubated for 10 h at 26°C, then washed with washing buffer, followed by washing four to five times using the elution buffer (10 mM reduced glutathione, 50 mM Tris–HCl, pH = 8). Subsequently, GST and GST-LcSVP2 protein were incubated with MBP-LcSMOS1 protein in 1 mL PBS solution at 4°C for 4 h, respectively. Then 50 μL GST resin was added and incubated at 4°C for 8 h, and GST wash buffer was used for about five times to obtain the bound protein. Bound proteins were eluted with sodium dodecyl-sulfate polyacrylamide gel electrophoresis loading buffer, and anti-MBP and anti-GST antibodies (Sangon Biotech, Shanghai, China) were used for the analysis of the bound proteins.

### Virus-induced gene silencing assays

Virus-induced gene silencing (VIGS)-mediated suppression of *LcSVP2* and *LcSMOS1* in litchi was performed according to the previous method [65]. Briefly, about 600 bp CDS fragment of *LcSVP2* and *LcSMOS1* were inserted into TRV2 to generate the pTRV2–LcSVP2 and pTRV2–LcSMOS1 fusion constructs. The empty vector pTRV1 and fusion vector pTRV2–LcSVP2/pTRV2–LcSMOS1 plasmids were separately transformed into *A. tumefaciens* GV3101. The pTRV1 and pTRV2–LcSVP2/pTRV2–LcSMOS1 agrobacterium cells were mixed with MES buffer (10 mM MgCl_2_, 10 mM MES and 200 μM acetosyringone, pH 5.6), then the infection solution pTRV1 and pTRV2 or pTRV2–LcSVP2/pTRV2–LcSMOS1 were mixed in a volume ratio of 1:1, and the empty vectors pTRV1 and pTRV2 were used as the negative control. The terminal buds of litchi at early Stage 3 were used for pTRV2–LcSVP2 and Stage 1 was used for pTRV2-LcSMOS1 infection transformation via stem injection at 6 mm below the terminal bud and submerging the terminal bud with the infection mixture. Twenty-one days later, terminal buds were analyzed with qRT-PCR for genes expression, and the VIGS groups with low *LcSVP2* and *LcSMOS1* expression were used for further analyses. Bud morphology was recorded with photos and the number of breaking terminal buds was recorded.

### Transient transformation assay in litchi terminal buds

The transient overexpression experiment was performed by a previous study [69]. The terminal buds of litchi cv. Feizixiao at early Stage 3 were used for the treatment. Full-length CDS sequence of the *LcSVP2* was inserted into the pCAMBIA1301 vector and then transformed into *A. tumefaciens* strain GV3101 for the injection treatment using the *35S* promoter. *A. tumefaciens* cells carrying the LcSVP2 recombinant plasmid were mixed with MES buffer, and the terminal buds were infiltrated with injection solutions using a syringe needle. The empty pCAMBIA1301 was used as a negative control. Twenty-one days later, terminal buds were detected by qRT-PCR, and the overexpression transformed buds with high *LcSVP2* expression levels were used for further analyses. Bud morphology was recorded with photos and the number of breaking terminal buds was recorded.

### Determining ABA content and ethylene production rate in litchi terminal bud

About 0.5 g of frozen terminal litchi buds per sample was used for extraction and analysis for ABA content, which was measured according to Yang et al. [70] using a Finnigan TRACE GC–MS set. The measurements were carried out with three tree-based biological replicates.

Measurement of ethylene production rate of litchi terminal buds was carried out based on Cronje et al. [28]. A total of 10 to 20 terminal buds per sample were collected according to bud stages and immediately put into an 8 mL vial and sealed with a rubber cap for 4 h at room temperature. Then 1 mL of air sample from the vial was injected into a gas spectrum chromatographer (Shimadzu GC-17A, Kyoto, Japan). After the air sample was taken, fresh weight and number of the sealed buds in each vial was recorded and ethylene production rate was calculated. The measurements were carried out with at least four tree-based biological replicates.

### Statistical analysis

All the experiments were performed with at least three biological replicates. The significant differences were analyzed using the Student’s *t*-test or Tukey’s multiple range test with SPSS (version 19.0; IBM Corp., Armonk, NY USA).

## Supplementary Material

Web_Material_uhae150
