# Supplementary figures and images for: MIKC type MADS-box transcription factor LcSVP2 is involved in dormancy regulation of the terminal buds in evergreen perennial litchi (Litchi chinensis Sonn.)
Source: Hortic Res. 2024 May 28;11(7):uhae150. doi: 10.1093/hr/uhae150 (PMC11233856; doi:10.1093/hr/uhae150)

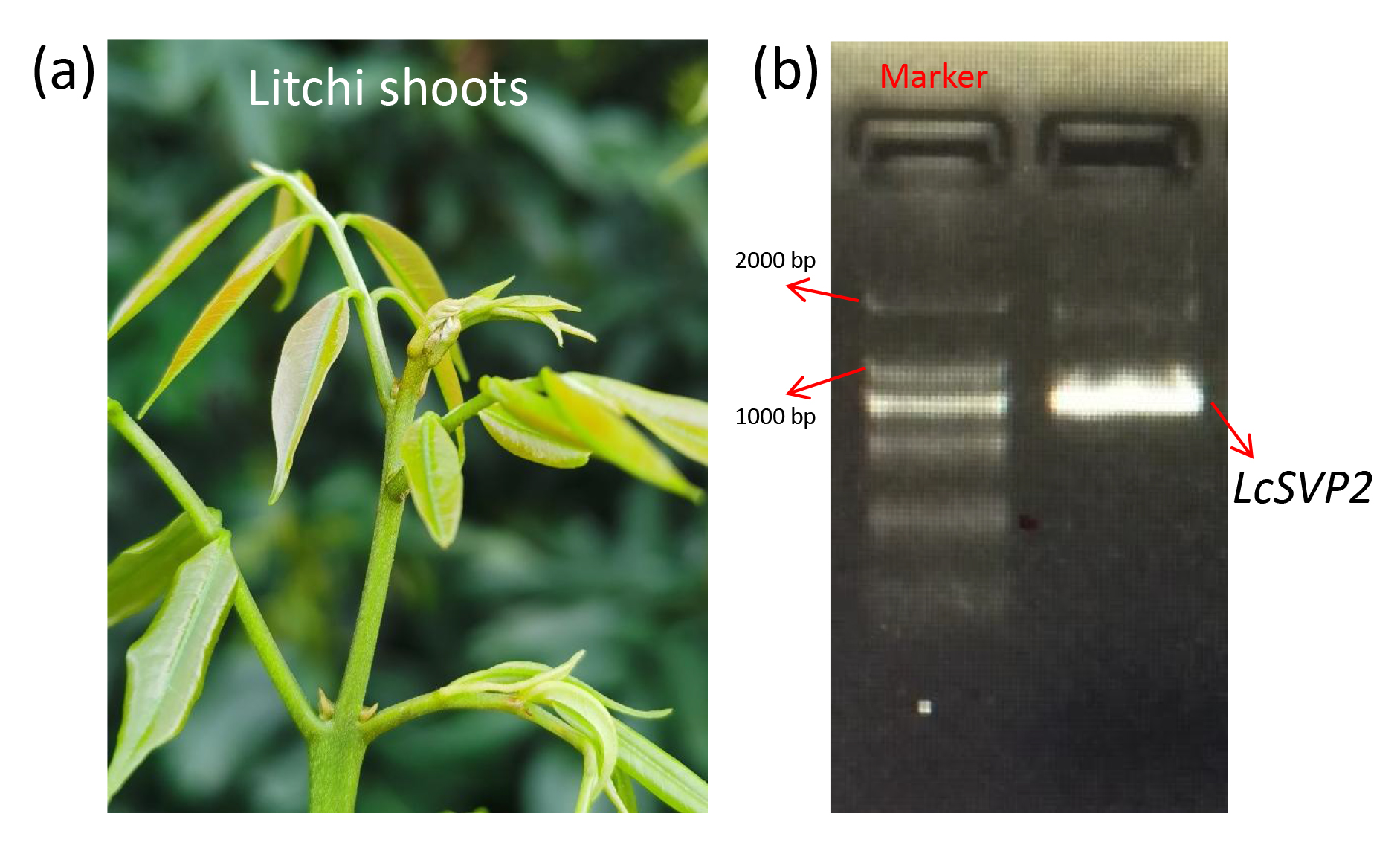

Supplement: Web_Material_uhae150 [file web_material_uhae150.zip › Figure S1.jpg]

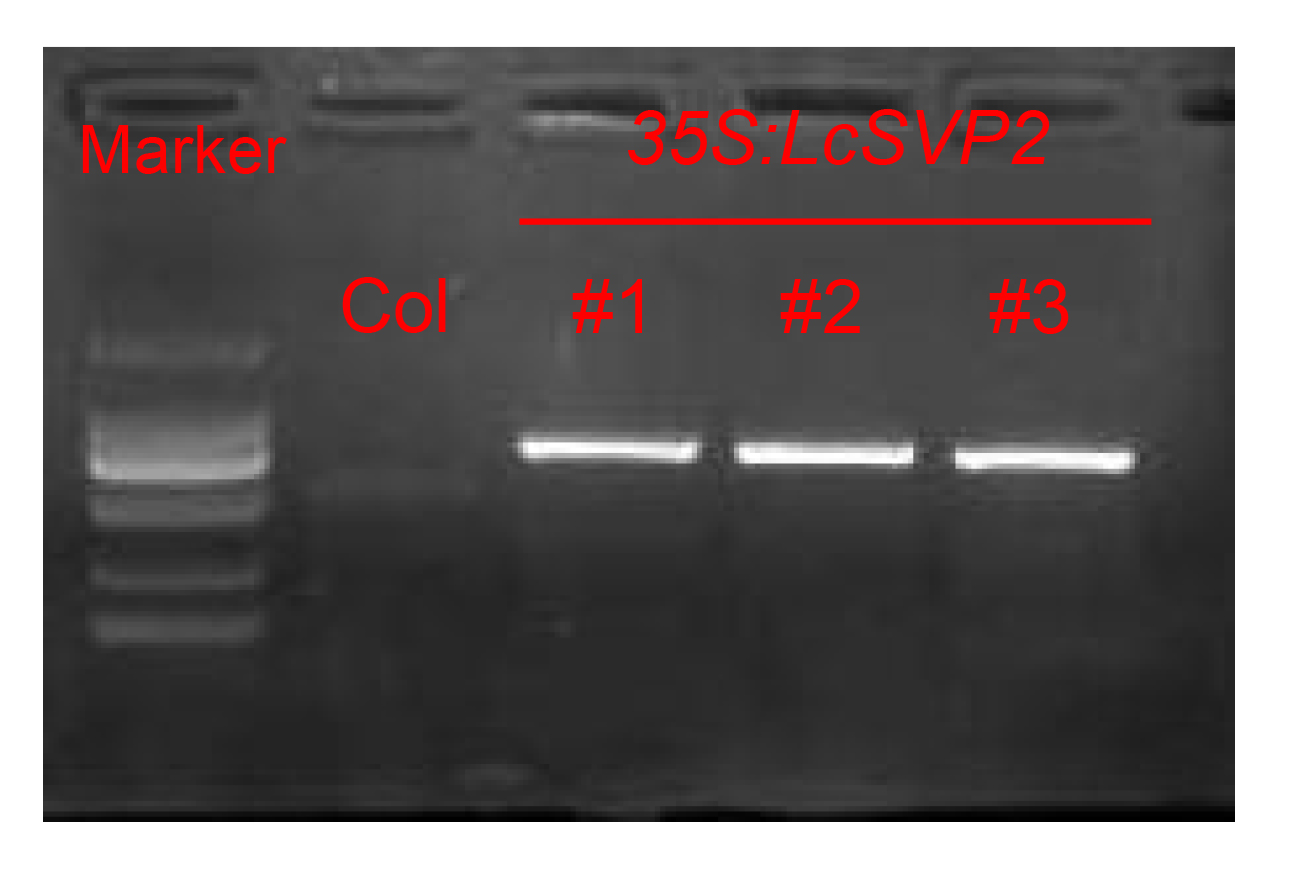

Supplement: Web_Material_uhae150 [file web_material_uhae150.zip › Figure S2.jpg]

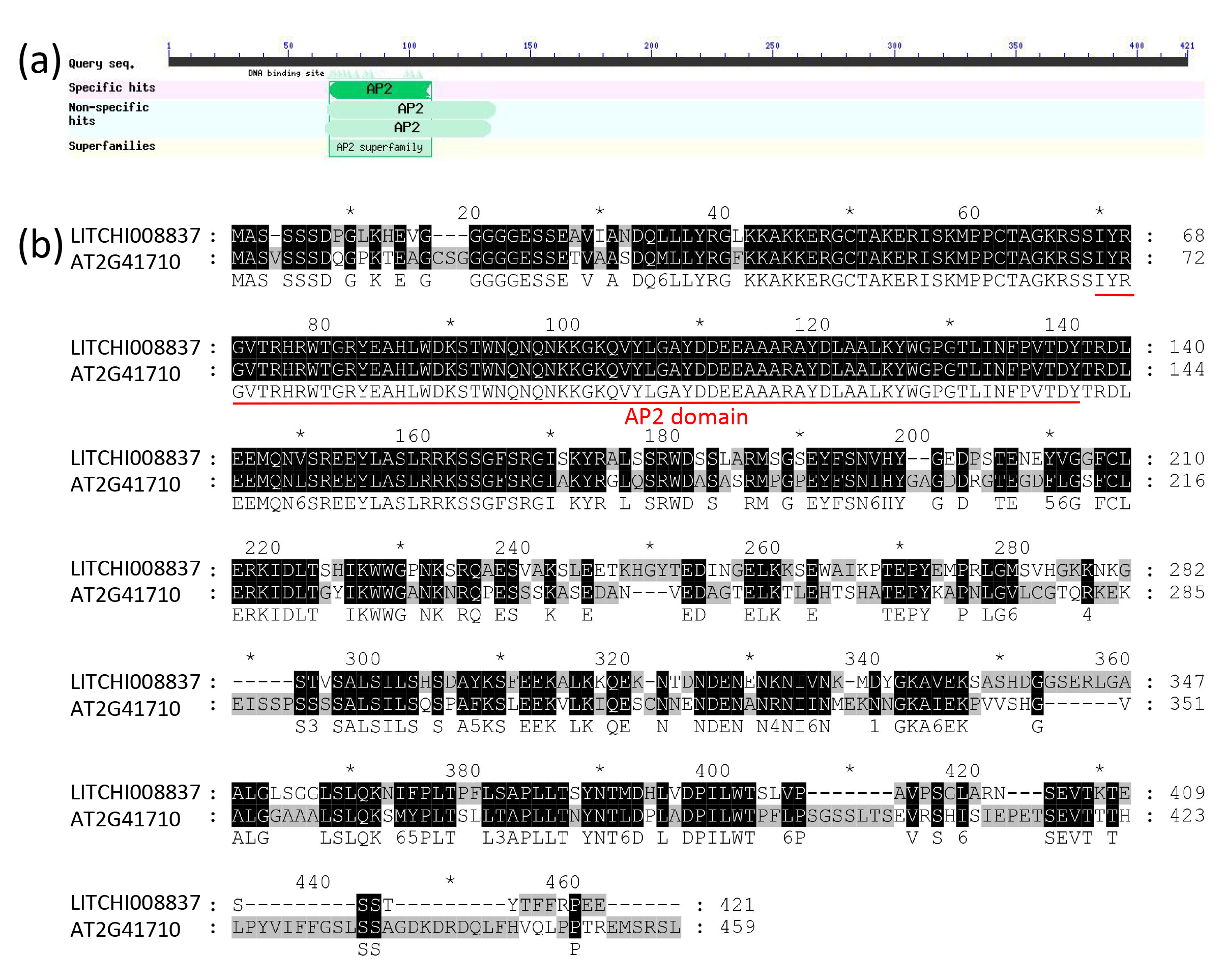

Supplement: Web_Material_uhae150 [file web_material_uhae150.zip › Figure S3.jpg]

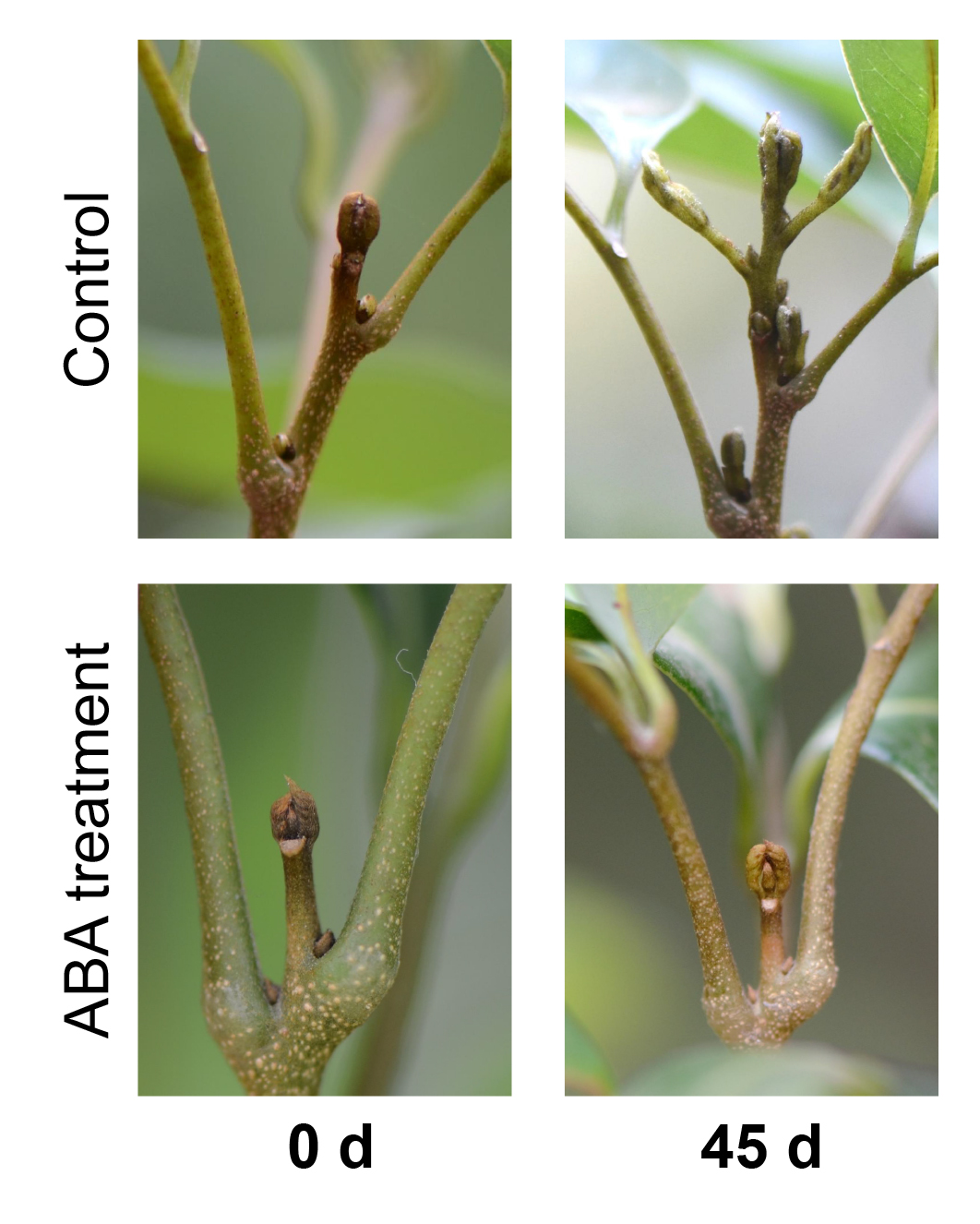

Supplement: Web_Material_uhae150 [file web_material_uhae150.zip › Figure S4.jpg]

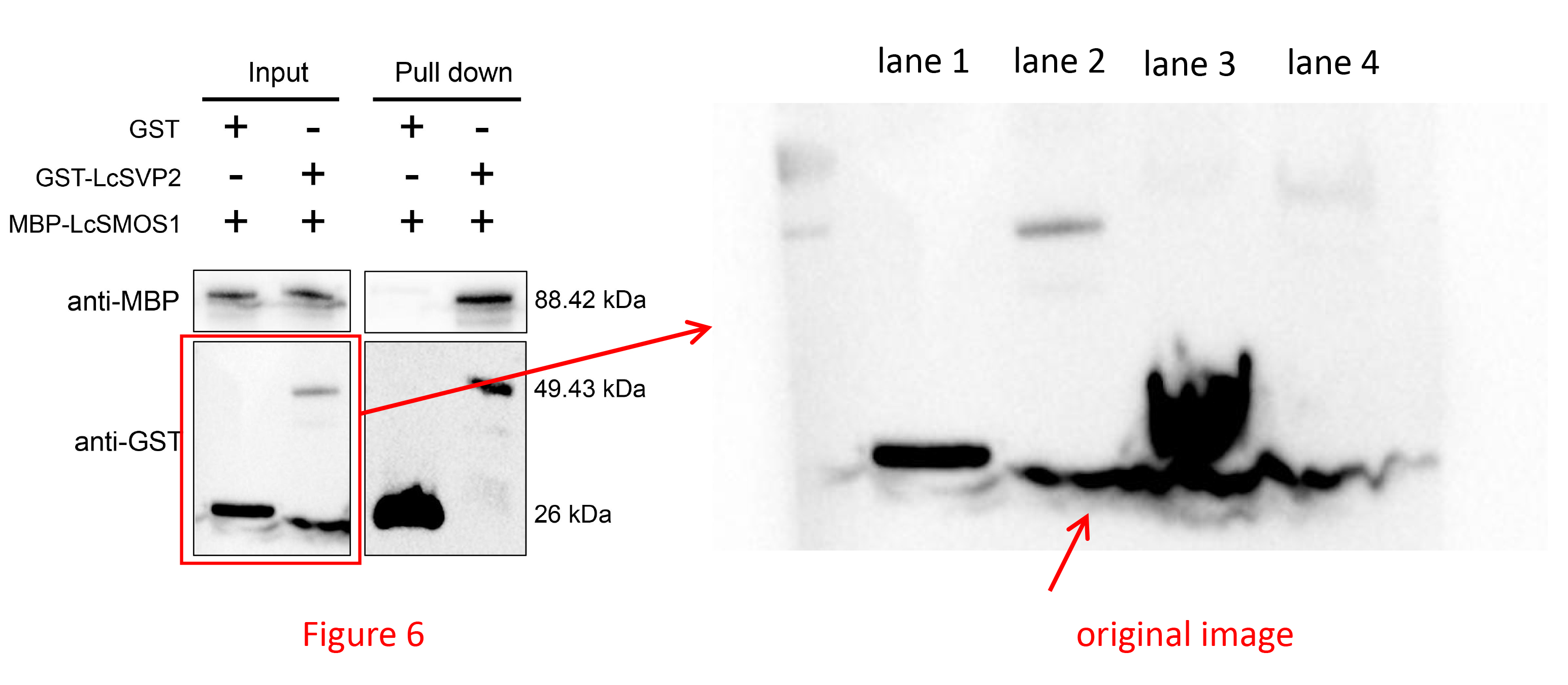

Supplement: Web_Material_uhae150 [file web_material_uhae150.zip › Supplementary data 1.jpg]
